# Identification of Salicylic Acid Mechanism against Leaf Blight Disease in *Oryza sativa* by SR-FTIR Microspectroscopic and Docking Studies

**DOI:** 10.3390/pathogens10060652

**Published:** 2021-05-24

**Authors:** Wannaporn Thepbandit, Narendra Kumar Papathoti, Jayasimha Rayalu Daddam, Kanjana Thumanu, Supatcharee Siriwong, Toan Le Thanh, Natthiya Buensanteai

**Affiliations:** 1 School of Crop Production Technology, Institute of Agricultural Technology, Suranaree University of Technology, Nakhon Ratchasima 30000, Thailand; w.thepbandit@gmail.com (W.T.); Narendrakumar.papathoti@gmail.com (N.K.P.); 2 Department of Animal Science, Agriculture Research Organization, Volcani Center, Rishon Lezion 7505101, Israel; jayasimharayalu@gmail.com; 3 Synchrotron Light Research Institute, Nakhon Ratchasima 30000, Thailand; kthumanu@gmail.com (K.T.); supatcharee@slri.or.th (S.S.); 4 Department of Plant Protection, Can Tho University, Can Tho City 900000, Vietnam; lttoan@ctu.edu.vn

**Keywords:** salicylic acid, rice bacterial leaf blight disease, pathogenesis-related (PR1b), induced resistance, SR-Fourier transform infrared microspectroscopy, docking studies

## Abstract

The present study was to investigate the application and mechanism of salicylic acid (SA) as SA-Ricemate for the control of leaf blight disease using a Synchrotron Radiation-based Fourier-Transform Infra-Red (SR-FTIR) microspectroscopy and docking studies. After treating rice plants cv. KDML 105 with SA-Ricemate, the leaves were inoculated with *Xanthomonas oryzae* pv. *oryzae*, the causal agent of leaf blight, and disease severity were assessed. The leaves were also used to detect changes in endogenous SA content. The results indicated that SA-Ricemate, as an activated compound, reduced disease severity by 60% at three weeks post-inoculation and increased endogenous content by 50%. The SR-FTIR analysis of changes in the mesophyll of leaves (treated and untreated) showed that the groups of lipids, pectins, and proteins amide I and amide II occurred at higher values, and polysaccharides were shown at lower values in treated compared to untreated. Besides, docking studies were used to model a three-dimensional structure for Pathogenesis-related (PR1b) protein and further identify its interaction with SA. The results showed that ASP28, ARG31, LEU32, GLN97, and ALA93 are important residues that have strong hydrogen bonds with SA. The docking results showed that SA has a good interaction, confirming its role in expression.

## 1. Introduction

Leaf blight of rice (LB) is a serious disease caused by *Xanthomonas oryzae* pv. *oryzae* (*Xoo*) that causes 20–50% losses in rice production worldwide [1,2]. It is regarded as one of the most devastating rice diseases in Thailand due to favorable climate conditions for pathogen survival and pathogenesis. The *Xoo* typically penetrates rice leaves through hydathodes at the leaf margin [3]. In addition, *Xoo* could access to xylem through wounds or openings of rice leaf sheath [4]. After going inside leaves, bacteria multiply in intercellular spaces, then enter plant cells and spread into the rice plant through its xylem [3]. Inside xylem, *Xoo* interacts with parenchyma cells [5]. The pathogen not only moves vertically through the leaf through primary veins but also progresses laterally through commissural veins [3]. After a few days, bacterial cells and EPS can fill the xylem vessels, then ooze out from leaf hydathodes, forming bacterial beads or strands on the leaf surface [4]. Plants use a salicylic acid-mediated response in pathogen attack, which is mediated by the PR protein and is effective in disease resistance [6].

Chemical pesticides, particularly copper hydroxide, have been recommended and widely used to control the disease. Nonetheless, its application is frequently ineffective and can harm humans and environment. In recent years, abiotic elicitors have been widely used to reduce the severity of plant diseases through mode of induced resistance (IR) in various crops, such as rice, chili, grapevine, chickpea, tomato, and maize [7,8,9,10,11]. IR is a type of plant defense that contributes to increasing plant resistance against pathogens by multiple mechanisms that can be induced by elicitors [12]. The priming of plants to increase defense metabolite expression in response to bacterial infection is an important feature of IR [13]. Secondary metabolites are substances formed as a result of interactions between plants and pathogens. The presence of phenolic compounds, terpenes, sulfur-containing, and nitrogen-containing secondary metabolites characterize these interactions [14,15]. Salicylic acid (SA) has been used as an elicitor against LB disease and acts as a signal molecule in plants by activating the expression of plant pathogenesis-related (PR) genes from the cinnamate pathway, which provides precursors for various phenylpropanoid compounds [15]. Disease resistance in rice plants can be activated by spraying with SA, resulting in a 38% reduction in LB disease severity [16]. Synchrotron Fourier-Transform Infra-Red (SR-FTIR) microspectroscopy has been developed as a novel bio-analytical technique that examines samples in a non-destructive manner [17,18]. This novel technique employs synchrotron light, which has small and bright characteristics and can identify molecular chemistry in biological tissues, including structural and non-structural lignin, proteins, lipids, carbohydrates, and their ratios [17,19]. For example, the vibration peaks 1700–1600 are referred to as Amide I, 1600–1500 as Amide II, and 1300–1200 as stretching hemicelluloses and lignins [19]. Therefore, SR-FTIR can help to track biochemical changes in plant tissues. Pathogens and pathophysiological defense mechanisms can both induce a wide range of defense-related pathophysiological (PATH) and antimicrobial (AMP) molecules. This system allows plants to respond quickly to environmental and other stresses, and it has long been studied as a resistance strategy used by the innate immune system, specifically for them, such as resistance response signaling pathways used by the plant cytoskeleton [9]. Even though many PR proteins and peptides have already been isolated, their current functional roles remain unknown. However, new scientific tools have assisted scientists in isolating and identifying a large number of new PR molecules. Previous research revealed that PR genes improve both biotic and abiotic stress tolerance, making them one of the most promising candidates for new stress-tolerant crop varieties. As a result, plant genetic engineering techniques, in general, are thought to be more interesting in the attempt to develop new disease-resistant transgenic crops that use PR genes than the specific knowledge of how PR genes can be used to do so. Many pathogens can now be dealt with by producing more defense enzymes at the same time [9]. The interaction of SA on rice plants against *Xoo*, reported that PR1b played a crucial role in enhancing disease resistance [20]. Similarly, PR1b also an important protein in local and systemic tissues during the interaction with SA on tomato against bacterium *Xanthomonas campestris* pv. *vesicatoria* [21]. The expression of protein PR1b with SA was surveyed on many types of research but underlaid their mechanisms [20,21]. However, there is no model of PR1b, especially the active site of PR1b on interaction to the systemic signal of SA during induced systemic resistance. In present in silico study as an in-depth appendix external to the design, due, on the bases of literature data [20,21] the PR1b proteins interaction with SA involved during induced systemic resistance evaluated, for better to understand the mechanism. The structure-function relationship of SA and pathogenesis-related (PR1b) was studied using molecular phylogeny, comparative modeling, and molecular dynamics (MD) to understand the evolution and viability of pathogen resistance. The purpose of this study was to characterize the mechanism of resistance by SA against LB disease in rice through in vitro and in silico studies using the SR-FTIR microspectroscopy technique and monitoring biochemical changes involved in plant defense mechanisms. Generally, gene expression, protein level, and histopathological response studies were used to characterize induced systemic resistance mechanisms, but the current study using SR-FTIR along with docking studies was novel.

## 2. Results

### 2.1. The Effectiveness of the SA-Ricemate Elicitor on Inducing Resistance against LB

The application of SA-Ricemate was firstly checked for the control of LB disease through a range of SA-Ricemate concentrations including 50, 100, 150, 200, 250, and 300 ppm. By spraying the SA-Ricemate elicitor three times before inoculating *Xoo*, the results of this experiment confirmed the control effectiveness of SA-Ricemate against LB. The results showed that 100 ppm or higher of SA-Ricemate elicitor showed a reduction of disease severity at 41–67% approximate control effectiveness calculated from disease severity, which was significant when compared to untreated and non-significant when compared to the positive control (57–58%) as commercial elicitor. In contrast, a 50 ppm SA-Ricemate elicitor dose only reduced the disease severity by 12–26% which is significantly lower than the positive control (Table 1). Therefore, ≥100 ppm SA-Ricemate elicitor doses showed high efficiency to control LB lesions on rice leaves.

### 2.2. Accumulation of Endogenous SA Content

The effect of exogenous application of SA-Ricemate on endogenous SA content in rice plants was evaluated. The results showed that all treatments increased the endogenous SA content in rice plants at 24 h post-inoculation, as shown in Table 2. The endogenous SA shown 30.78, 51.27, 48.00, 54.05, 48.84, 51.44, and 38.33%, respectively with treated with different concentration of Ricemate, compared with control (11.40%). This is considered a significant difference when compared to the copper hydroxide treatment, which had an endogenous SA increase of 5.19% versus 11.40% in the untreated samples.

### 2.3. SR-FTIR Microspectroscopy

**Figure 1:**
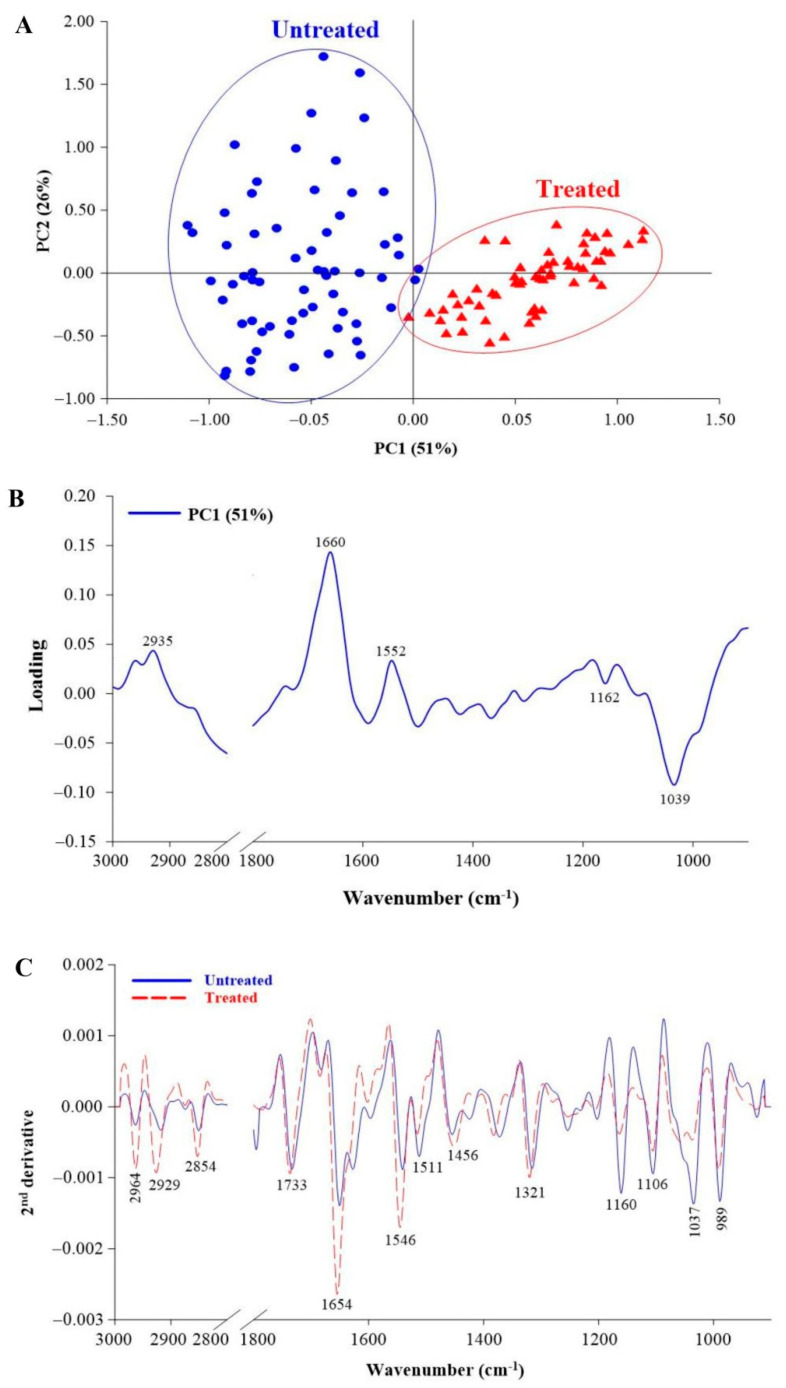
The SR-FTIR spectra of mesophyll of rice leaf tissue; (**A**) Principle component analysis (PCA) analysis of a rice leaf, (**B**) loading plots from PCA analysis of mesophyll treated and untreated group, and (**C**) Overlay of the average 2nd derivative spectrum range of 3000–2800 cm^−1^ and 1800–900 cm^−1^ of mesophyll of rice leaf tissue between treated with SA-Ricemate compared to the untreated and then challenge inoculation with *Xanthomonas oryzae* pv. *oryzae*.

The SR-FTIR spectra are used for investigating changes in rice biochemical and cellular compositions after treating with SA-Ricemate. The results show a clear difference between the untreated (water-mock) and treated clusters of 100 ppm SA-Ricemate, as shown by the PCA score plot, which was explained by 51% PC1 and 26% PC2. (Figure 1A). PC1 had a high positive loading at 2935, 1660, and 1552 cm^−^^1^, which corresponded to the positive score plot from the treated sample. Whereas, the high negative loading from the PC1 at 1162 and 1039 cm^−1^ corresponded with the negative score plot from the untreated sample (Figure 1B). The difference in the SR-FTIR spectra changes in the mesophyll revealed three distinguishable regions in the SA-Ricemate treated sample (Figure 1C). The first region (3000–2800 cm^−^^1^) of the treated sample was higher than the untreated sample, according to the CH2, CH3 from lipid groups, with a clear peak at 2964, 2929, and 2854 cm^−^^1^. The second region (1700–1500 cm^−^^1^) is composed of proteins and peptides with an amide group, and the treated sample has a peak at 1654 and 1546 cm^−^^1^ that is higher than the untreated sample. The third region (1300–900 cm^−^^1^) involving polysaccharides and carbohydrates has untreated sample peaks at 1160, 1106, 1037, and 989 cm^−^^1^ that are higher than the treated sample peaks at 1160, 1106, 1037, and 989 cm^−^^1^.

### 2.4. Homology Modeling of PR1b Domain

The lack of a three-dimensional structure of PR1b in the database, as well as the importance of PR1b structure in studying interactions with SA, prompted to create a PR1b model. A model for PR1b was predicted by using homology modeling methods and this structure was used for docking with SA in this study. The protein sequence was retrieved from the UNIPROT database and compared to a template structure.

### 2.5. Identification of Template

Only ICFE A (Chain A, *Solanum Lycopersicum*) showed a high degree of sequence identity with the PR1b domain when analyzed using BLAST. In this case, 1CFE was chosen as a template structure, and the structure was collected using the PDB database. This comparison revealed that the target sequence and the template had a sequence identity of 56%. Alignment of the target sequence and template structure should be established at a high level of identity.

### 2.6. Sequence Alignment

**Figure 2:**
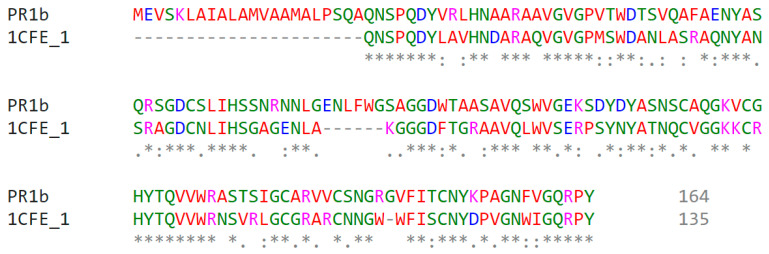
Sequence alignment of PR1b with template 1CFE. * indicates amino acids from PR1b and 1CFE are same in comparison whereas . and : indicates amino acids have some similarity regarding groups in comparing with PR1b and 1 CFE. Gaps in the alignment was shown by -----.

To calculate comparative protein models, a precise similarity relationship between the target protein and its template structure is required. In homology modeling, a template structure with at least 30% identity to PR1b was required. The protein sequences for PR1b and the protein on which the alignment was performed show that the two sequences share 50.4% identity (Figure 2).

### 2.7. Prediction of the Three-Dimensional Structure

**Figure 3:**
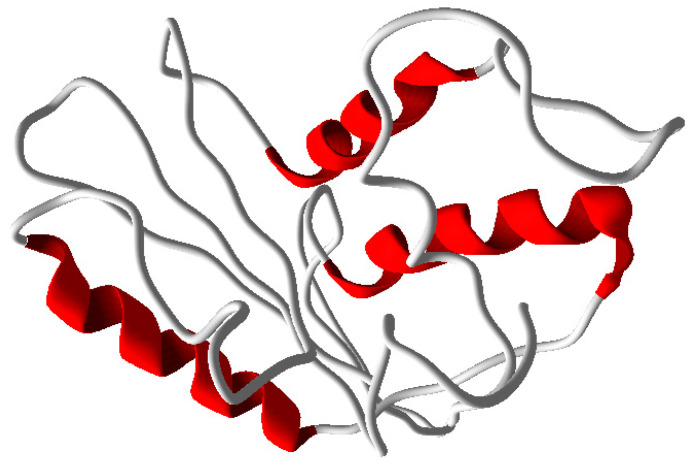
Modeled structure of PR1b with 3 helices.

MODELLER9V7 was used to create the three-dimensional model, and the final model was used for validation and subsequent processes after energy minimization. There were three helixes and no sheets in the quaternary structure (Figure 3).

### 2.8. Structural Validation

**Figure 4:**
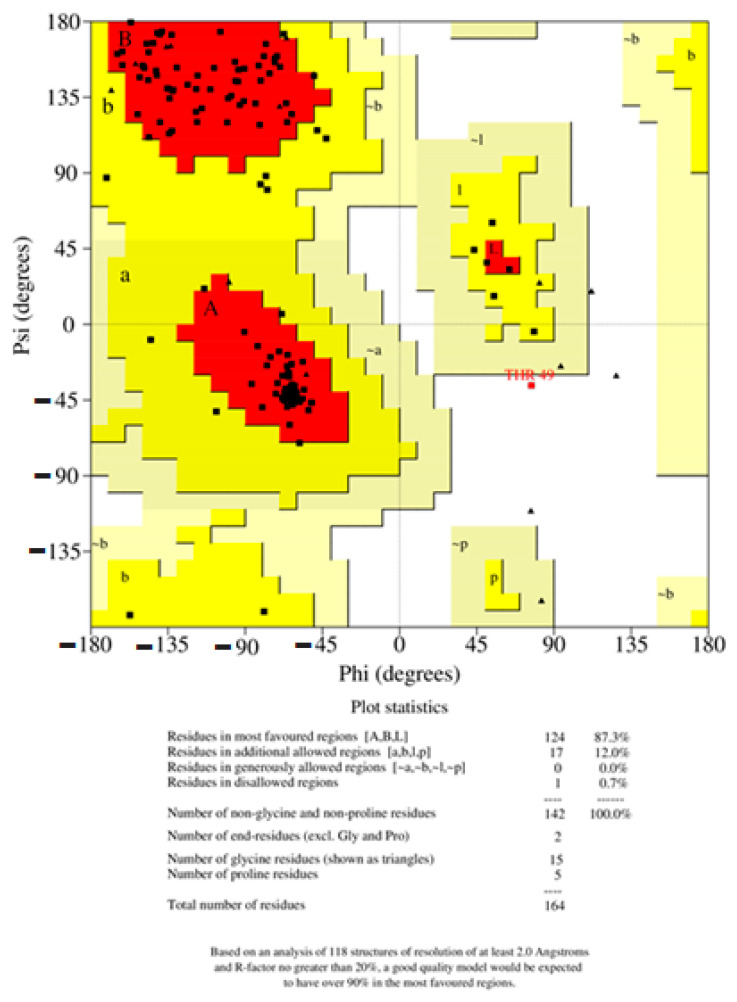
Ramachandran Plot analysis of PR1b predicted model.

**Figure 5:**
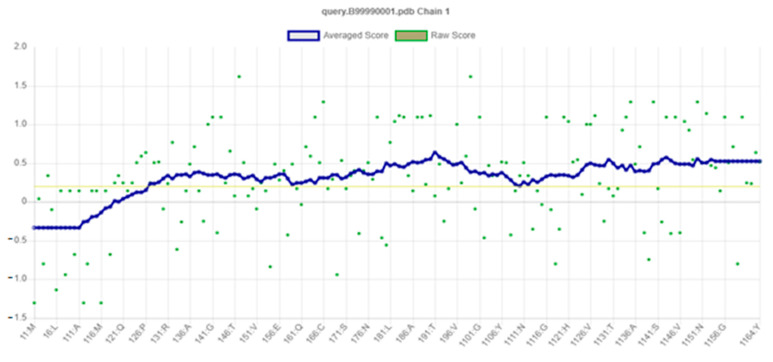
The 3D profile of PR1b using Verify_3D server; overall quality score indicates residues is reasonably folded.

**Figure 6:**
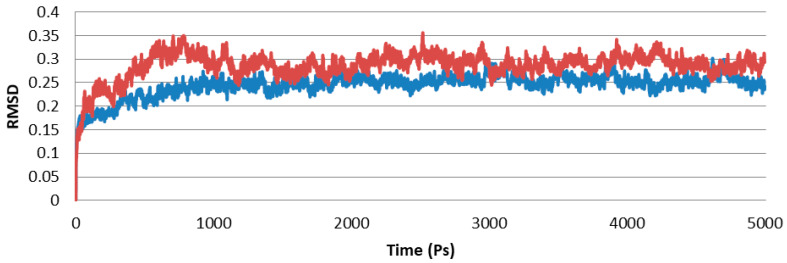
Calculated RMSD graph of PR1b.

The structural model of PR1b of *Oryza sativa* L. was examined with a variety of structural variables to validate its results of the psi and phi distributions from the Ramachandran plot. The results showed that 95.3% of each PR1b are in the preferred region and 2.0% are in the generously permitted regions (Figure 4). Procheck was applied to verify the generated structure against static analysis, and PRO-MOD was used to evaluate the generated structure, were analyzed by PROCHECK (Figure 5). The preferred range of the basic-protein motifs present in this region suggests that the predicted model of PR1b is reliable. Mollify deviation was used to find the differences between the calculated and predicted molecular structures, a molecular dynamics simulation was employed. The templates and backbones were on average 0.25° out of flexion and 0.3° of extension concerning the Cα were 0.25° to 3.0° (Figure 6).

### 2.9. Active Site Identification

**Figure 7:**
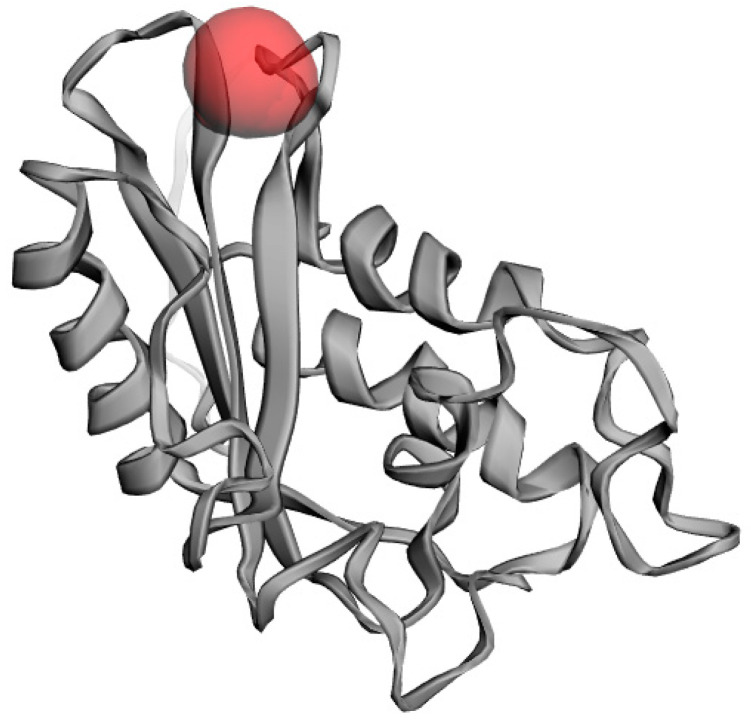
Representing active site Pockets of the PR1b shows the highest area and volume (red color).

Predicted model binding locations were identified by the CASTp server for docking purposes. This PR1b model had no previously considered structure; however, based on the predicted model, the largest pocket size and important residues involved in the active site were predicted to dock with SA. The abundance of amino acid residues in the active site of PR1b showed that the structure is highly conserved with the template, and the residues involved in the active site are ASP28, ARG31, LEU32, GLN97, and ALA93 (Figure 7).

### 2.10. Docking Studies of SA with PR1b

**Figure 8:**
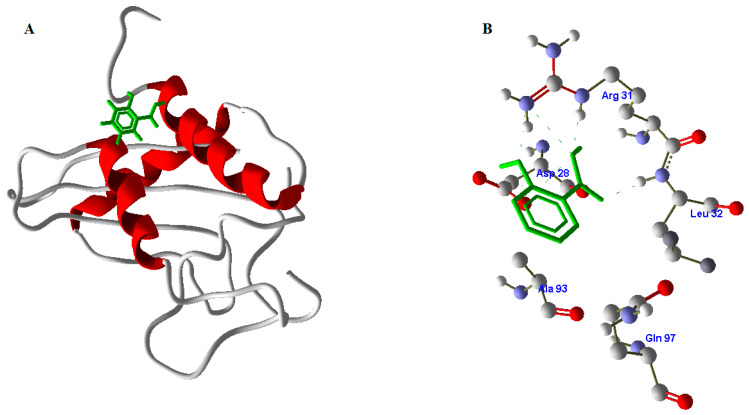
Docking studies of SA with PR1b. (**A**) Molecular interaction of SA (green color) with PR1b (red color). (**B**) Amino acids from PR1b involved in docking to SA (green color). Hydrogen bonding interactions are mentioned in green color lines.

Salicylic acid was prepared on scaffolds with a larger radius of expansion. Previous research has shown that docking is the best strategy for demonstrating ligand-protein interactions and protein-compound binding from docking results. According to the docking results, the PR1b residues play an important structural and functional role. Docking studies confirmed that SA has a good interaction with PR1b. SA formed hydrogen bonds with the active residues of PR1b, confirming its role in enhancing PR1b’s function in pathogenicity protection. The docking score generated by the interaction was 26.2 KJ/mole, and Figure 8 depicts the complex of PR1b and SA.

## 3. Discussion

Pathogen activation of plant defense reactions may employ a variety of signaling mechanisms, the most effective of which is endogenous SAagainst pathogens. The abiotic elicitor SA-Ricemate, which is based on the plant defense-elicitor SA, was described in this study as a potential new bio-stimulant that acts as an elicitor of resistance. The concentration of SA-Ricemate determines the efficacy of the treatment in controlling the LB disease, with studies demonstrating that a concentration range of 100 ppm to 300 ppm is an optimal concentration range. Low concentrations of less than 100 ppm are thought to be ineffective against rice LB. This could be because the appropriated concentration of elicitor has an efficient plant defense mechanism. Several studies have previously recognized the role of SA in plant defense response and involvement in endogenous signal-mediated local and systemic plant defense. According to War et al. (2011), chickpea (*Cicer arietinum* L.) responded to a SA treatment at 1.5 mM with greater induction of plant defense enzymes, such as POD, PPO, H_2_O_2_, and defense protein activities, than the use of SA at 1 and 2 mM. These findings suggest that SA at 1.5 mM could be effective for activating the plant immune system [19]. Wani et al. (2017) reported that applying SA to plants improved the initiation of pathogenesis-related gene expression, as well as the synthesis of defensive compounds involved in local and systemic acquired resistance [22]. Yang et al. (2019) found that pretreatment with SA resulted in lower rice blast disease (*Magnaporthe oryzae*) and higher expression levels of rice defense-related genes PR1a, PAL, HSP90, and PR5 on rice leaves [23].

Through a signaling transduction pathway, the inducing resistance (IR) mechanism protects distant parts of rice plants. The SA pathway is a major signaling pathway during rice LB resistance by crosstalk between signaling that can provide a potential for efficient energy and accumulation of potential enzymes against pathogens [24,25,26]. According to Sticher et al. (1997), the SA signaling pathway can be triggered by exogenous SA, which increases disease resistance, because this pathway is related to systemic acquired resistance (SAR), which can occur when endogenous SA accumulates and is activated after plant pathogen infection. Our findings show that the accumulation of endogenous SA content in treated rice plants after *Xoo* infection is approximately 50% higher than in non-treated rice plants. The results of this research are in line with previous studies. After treatment with the elicitor, ASM 1 mM for Tomato Virus and Citrus Viroid, an increased concentration of endogenous SA was observed in tomato and citrus plants [26]. Babu et al. (2003) discovered in their study that a pre-treatment with SA at 1000 µmol L^−1^ by a significant increase of 44.35% of endogenous SA accumulation resulted in a significant reduction of LB development and LB lesion length of around 20% in the susceptible rice variety IR50. Thus, the concentration of endogenous SA may be an important component of this resistant mechanism in rice, determining immune activation selectively during pathogen infection and invasion [27,28].

Endogenous SA or resistance signal of SA is involved in the synthesis of secondary plant defense metabolites such as terpenes, phenolic compounds, and alkaloids [29,30]. The concentration of endogenous SA can influence the selective activation of defense responses during pathogen infection and invasion, which can alter the plant’s physiological, biochemical, and molecular levels [15,19]. SA activates several enzymes, including peroxidase (POD, POX), superoxide dismutase (SOD), polyphenol oxidase (PPO), and phenylalanine ammonia-lyase (PAL) [31]. These enzymes protect the cell from oxidative stress and play an active role in metabolism [19,29]. These plant defense metabolites and enzymes take part in creating physical barriers at biochemical and cellular levels on host plants. The changes at the biochemical and cellular level of plant tissues could be characterized by SR-FTIR spectroscopy. Principal Component Analysis (PCA) score plot is a multivariate technique used to decompose the data matrix and to concentrate the source of variability of the data into loading PCs. In this work, a PCA score plot was used to visualize the existing clusters from the sample data. Spectra analysis of treated and untreated samples was performed in the spectral range of 3000–2800 cm^−1^ and 1800–900 cm^−1^. The lipid region was found at 2800–3000 cm^−1^ which is assigned to asymmetric/symmetric of CH3/CH2. The spectral band at 1733 cm^−1^ arises from the C=O ester of pectin. The region at 1515 cm^−1^ englobes the stretching vibration of C=C aromatic skeleton vibration from lignin. In addition, the region between 1200–900 cm^−1^ is induced by functional groups of C-O-C glycoside either mainly composed of hemicelluloses or by polysaccharides. The PCA score plot (Figure 1A) can be separated by a total variance of 77% from PC1 (51%) and PC2 (26%). The clear separation of 51% from PC1 showed the relative distinction which indicated the chemical composition between groups of samples that corresponded to loading. The positive loading PC1 was the one that most contributed to treated samples that present higher intensity of amide I protein and lipids. The negative loading PC1 classified untreated samples that corresponded to the polysaccharide region (Figure 1B). The average second derivative spectrum (Figure 1C) presented high intensity at the main peak which is correlated to loading. The spectral data from the treated samples showed higher intensity of lipids and proteins than the untreated samples data whereas the high absorbance intensity of the polysaccharide region was higher in untreated samples data.

The association of the biomolecular and its intensity from the average spectra suggests higher accumulations of lipids (3000–2800 cm^−1^) and proteins (1700–1500 cm^−1^) [31]. Lipids are the major component of a cell membrane, and they play a role in several cellular systems, including energy storage, protection communication, structural support, and hydrocarbon as a monomer that prevents water loss, protects plant cells and nutrients, and coats the surface of plant leaves to inhibit pathogens. According to Zhang et al. (2015), phospholipids and phosphatidic acid (PA) belonging to the membrane lipid bilayer act as a signaling immunity and link to ROS activity and SA accumulation, whereas Gao et al. (2017) reported lipids and lipid metabolites are important in rice plants to protect against LB and rice blast via plant-microbe interactions [32,33]. Furthermore, enzymes such as beta-glucanases and chitinases are components of pathogenesis-related (PR) proteins, which play an important role in protecting plant cells from pathogen infection [17,32,33,34]. When plants detect an insect, fungal, bacterial, or virus-viroid attack, beta-1,3-glucanase or chitinase activates plant defenses against fungal infection. Anita et al. (2014) reported that systemic induce resistance in rice against rice root-knot can occur by increasing chitinase enzyme activity and plant defense protein [35,36,37]. Amide I and amide II are linked to a second protein, which is an important amino acid implicated in disease resistance, such as L-phenylalanine, which is a precursor to plant defense metabolites via the phenylpropanoid and lignin pathways. According to Macoy et al. (2015), several plant amide groups, such as hydroxycinnamic acid amides, have shown significant interaction between plant and pathogen [38,39,40]. Similarly, Thamunu et al. (2017) reported changes in absorbance as a peak of proteins (1656 cm^−1^) shifted to higher by induced resistance with *Bacillus subtilis* strain D604 on chili plants, indicating the response mechanisms against anthracnose disease infection [36]. These biochemical changes have been linked to callose deposition and cell wall thickening in plants. Furthermore, polysaccharides (1200–900 cm^−1^) as carbohydrates or sugars groups are required to supply the energy source for defenses’ and can be used as regulation signals for defense genes, which may be useful in controlling plant diseases [41,42]. During infection, plants modify or change their sugar source and activate their defense responses by increasing PR proteins and some sugars used as activating agents to combat pathogens [43,44].

Protein structure prediction is the most reliable and most widely accepted technique. Conformational modeling provides the sequence identities for one or more target proteins [45]. If the sequence identity between the template and the target protein is significant, the structure-based drug design approach can even produce a correct model. Predicted homology was performed using a template-based 3D model of the structural coordinates, while modeling and refinement were performed with MODELLER 9V7 using software-specified line commands [46]. Secondary structure analysis of developed PR1b from *Oryza sativa* predicted that there is an expected similarity in the abundance of α-helix, β-sheet, and other secondary conformations of the structural β-sheet relative proportions of the model PR1b [47,48,49,50]. To be more specific, PROCHECK and verify 3D checked out and verified a very simple and accurate model that correctly predicted the final structure [51,52]. Because there was a difference in the RMS deviation of this structure from the template structure, as well as a difference after MD simulations, our predicted form can be used safely for docking [53,54,55,56]. Because the PR1b sequence and structure of both proteins are conserved, the function of the respective proteins is highly likely to be the same. These are conserved during all of the commoner’s expansion of plant lectins [57,58,59]. They discovered that all lectins share a common active site that is filled with highly conserved amino acid residues, as well as one that varies by species [60,61,62,63]. The calculations include changes in molecular weight and binding enthalpy [64]. Lipinski’s Rule Five docking theory confirmed that the SA-designed molecule passed completely without issue under the Salicylic docking standard. PR1b and Salic acid react to broaden and unfold the protein, which appears to be appropriate for deducing domain and compound binding mechanisms in docking studies.

## 4. Materials and Methods

### 4.1. Rice Cultivar

Thai jasmine rice variety KhaoDawk Mali 105 (*Oryza sativa* L.) was used in this research as a susceptible variety.

### 4.2. Xanthomonas oryzae pv. oryzae Strains and Culture Conditions

The virulent *Xoo* strain SUT1-121, causal agent of LB disease, was obtained from the stock culture of the Plant Pathology and Biopesticide Laboratory (PPB Lab), Suranaree University of Technology, Nakhon Ratchasima, Thailand. The bacterial strain *Xoo* was inoculated on nutrient glucose agar (NGA) media and incubated at 28 ± 1 °C, 180 rpm for 48 h. For preparing culture suspension, the *Xoo* bacteria were inoculated in nutrient glucose broth (NGB) and incubated at 28 ± 1 °C, 180 rpm for 48 h. The specific absorbance of the *Xoo* suspension was measured and adjusted at OD 0.2 in sterile distilled water to approximately 1 × 10^8^ CFU mL^−1^ [15,65,66].

### 4.3. Preparation of a Commercial Abiotic Elicitor Product (SA-Ricemate)

The exogenous SA elicitor (SA-Ricemate) prototype is a product of Bioactive Agro Industry Co. Ltd., Nakhon Ratchasima, Thailand. SA-Ricemate contains SA, which is the most commonly used product in Thailand. The SA concentration is appropriate for Thai rice which was developed at PPB Lab, Suranaree University of Technology, Thailand.

### 4.4. Efficacy of the SA-Ricemate Elicitor in Inducing Resistance Against LB

The efficacy of SA elicitor (SA-Ricemate) in inducing resistance against LB was studied at concentrations of 50, 100, 150, 200, 250, and 300 ppm. With five replications and two pots per replication, the treatments included a positive control (commercial elicitor and copper hydroxide 77% WP) and negative control (Water). Rice seeds cv. KDML 105 were soaked with sterile distilled water for 24 h before planting. The seedlings were transferred into 30 cm diameter plant pots that contained 5 kg of farm soil from the Suranaree University of Technology. The pots were kept under greenhouse conditions with 12 h of photoperiod, 28 ± 4 °C, and 60–75% of humidity. Sprays of SA-Ricemate elicitor at various concentrations and a control of 30 mL on rice leaf in each treatment were shown after 15, 30, and 45 days. At 50 days after sowing, the rice plants were inoculated with a density of *Xoo* suspension at 1 × 10^8^ CFU mL^−1^ on top-leaves by cutting the leaf 3 cm from the leaf tip, then covered with transparent bags and incubated for 24 h [67,68,69,70].

The severity of LB disease was recorded three times at 7 days post-inoculation (DPI) intervals using the International Rice Research Institute (IRR) disease score chart for assessing LB symptoms. Then, the percentage of disease severity was calculated by using the following, Formula (1).

(1) $$\mathrm{Disease}\;\mathrm{severity}\;\left(\%\right)=\left(\frac{\sum_{i=1}^{n}r_{i}}{n\;\times m}\right)\;\times \;100$$

where ‘r’ is the set of numerical ratings, ‘n’ is the total of evaluations per sample, and ‘m’ is the maximum value used for the evaluations [15,71]. The reduction of disease severity was calculated using the formulated Equation (2):

(2) $$\mathrm{Reduction}\;\mathrm{on}\;\mathrm{disease}\;\mathrm{severity}\;\left(\%\right)=\frac{\mathrm{DSn}-\mathrm{DSt}}{\mathrm{DSn}}\;\times \;100$$

where DSn is the calculated disease severity from untreated samples, and DSt is the calculated disease severity from elicitor-treated samples.

### 4.5. Determination of Endogenous SA

After soaking 0.5 g of rice leaf samples from each treatment in liquid N2, they were homogenized with 1 mL of extraction buffer (methanol: glacial acetic acid: water; 90:9:1 by volume) and centrifuged at 14,000× *g* for 10 min at 4 °C. In an equal volume, 0.5 mL of the supernatant above the sediment was mixed with 0.02 M ferric ammonium sulfate solution and incubated for 5 min at 30 °C. The absorbance at 530 nm was measured using a Bio-Tek, Winooski, VT, USA microplate reader and compared to standard references to determine the amount of endogenous SA in the sample [68,72].

### 4.6. Biochemical Change Analyses Using SR-FTIR Microspectroscopy

The leaf samples were chosen from the previous experiment’s best concentration treatment. The leaves were fixed with Optimal Cutting Temperature compound (Tissue-Trek^®^, Torrance, CA, USA), then rapidly cooled in liquid nitrogen. Each frozen sample was transversely cut with a cryostat microtome (Leica 3050 S, Wetzlar, Germany) at 7 microns and placed on 13 × 2 mm infrared-transparent barium fluoridate slides [15,34].

### 4.7. Data Analysis of SR-FTIR Microspectroscopy

The spectral data were collected and imaging was done at the beamline 4.1 IR Spectroscopy, Synchrotron Light Research Institute (SLRI). The determinations were carried out by using the mode of mapping with an aperture size of 10 × 10 μm, 4 cm^−1^ of spectral resolution, and 64 scans for the background [73,74,75]. Spectral derivative and equipment were performed by OPUS 7.2 software (Bruker Ltd., Hanau, Germany) then, data analyzed by cytospec^TM^ software and unscramblerX10.0 software, NJ, USA [34,75].

### 4.8. Domain Identification and Template Search

All molecular computations were done on the cutting-edge, using AMD 64-powered Linux dual-processing Linux workstation. No PR1b structure was found in the database, so the amino acid sequence of the pathogenesis-related protein from *Oryza sativa* L. (Uniprot ID: P04284) was retrieved from the UNIPROT databank. *PR1b* was submitted to the SBASE domain search server. Using FASTA format, the protein searched against the PDB data bank’s database in BLAST search to identify related proteins that have similar three-dimensional structures to the query [47,48]. Predicted protein structures were found by the BLAST (https://blast.ncbi.nlm.nih.gov/Blast.cgi (accessed on 25 January 2021)) against the protein database provided and which has the highest sequence identity to the protein PR1b and the results were highlighted as a preferred. Protein coordinates from the templates were assigned to the sequence using spatial requirements. ClustalX [50,51,52,53,54] uses the default parameters to align the reference structures to the target sequences.

### 4.9. D Model Building of PR1b

Homology modeling is used to create the initial structure of PR1b. The structure is generated using MODELLER9V7, which is further developed using molecular dynamics methods. The software generated fifty models, and the least energy of these was chosen for further analysis. The generated model was subsequently used to assist in the stabilization of the three-dimensional protein structure by molecular dynamics simulations [53,54]. The NAMD 2.8 was used to simulate generated structure using CHARMM fields. The tracking method is multiple-time stepping with long-range electrostatic determined after every two steps. This study employs Hamilton’s equations of motion to find new velocities. Data was used to produce the new model and compared it to its previously known template thermodynamic properties in terms of RMSD [55,56].

### 4.10. Structure Validation of PR1b

After an alternative structure was determined using molecular dynamics and environment simulation, a low Root Mean Square Deviation (RMSD) is generated, which is then evaluated using the PROCHECK and structure evaluation server for profile applications [57]. After PR1b is minimized, this protein can be used to identify the active site and docked with SA.

### 4.11. Active site Identification of PR1b

With the successful completion of the final model, structural comparison of the template and the model was performed to identify the binding sites of PR1b from *Oryza sativa* L. In addition, the binding sites were predicted using the CASTp server for the PR1b modeled structure [58]. This server was used to generate different binding pockets and their volumes, from which we selected the highest pocket in size and volume for docking with SA.

### 4.12. Docking Studies with SA

Docking studies were performed to gain insight into the binding conformation of SA using FRED (Open eye scientific software, Santa Fe, NM, USA) [57,58,59,60,61]. Because PR1b has multiple sites that are expected to react with different types of confirmations, and SA was expected to produce different confirmations with novel physiological properties, a library of different SA confirmations was built. To investigate the interaction of PR1b with SA, a strong correlation was discovered between docking values and experimental values [59,60].

## 5. Conclusions

The findings of this study showed that SA-Ricemate can reduce the severity of bacterial leaf blight on rice by inducing resistance. The resistance mechanism occurred through the expression of endogenous SA as the systemic signal, the alterations of lipids, pectins, proteins amide I, proteins amide II, and polysaccharides. Homology modeling was used to generate a three-dimensional structure for the PR1b sequence during IR. It was discovered in this study that ASP28, ARG31, LEU32, GLN97, GLN, and ALA 93 of PR1b form hydrogen bonds with SA. The docking studies shown that salicylic acid is involved in expression of PR1b mechanism.

## Figures and Tables

**Table 1:** Effectiveness of SA-Ricemate on the control of bacterial leaf blight disease of rice cv. KDML 105 caused by *Xanthomonas oryzae* pv. *oryzae* strain SUT1-121.

Treatment ^1^	Disease Severity (%) ^2^			Control Efficacy % (%)		
	7 DPI	14 DPI	21 DPI	7 DPI	14 DPI	21 DPI
SA-Ricemate 50 ppm	26.04 ± 2.75 c	35.42 ± 4.54 c	39.58 ± 2.75 c	13.80 ± 7.75 a	12.88 ± 4.96 a	26.77 ± 10.83 a
SA-Ricemate 100 ppm	17.71 ± 2.75 b	18.75 ± 1.04 b	21.88 ± 1.04 b	41.39 ± 4.81 b	53.88 ± 1.18 b	59.53 ± 3.91 b
SA-Ricemate 150 ppm	15.63 ± 3.12 b	16.67 ± 1.04 b	22.92 ± 1.52 b	48.28 ± 8.51 b	59.00 ± 3.12 b	57.60 ± 5.19 b
SA-Ricemate 200 ppm	16.67 ± 2.77 b	19.79 ± 2.11 b	21.88 ± 1.00 b	44.83 ± 7.39 b	51.31 ± 3.25 b	59.53 ± 2.99 b
SA-Ricemate 250 ppm	13.54 ± 2.71 b	17.71 ± 2.08 b	20.83 ± 2.18 b	55.18 ± 7.73 b	56.44 ± 3.49 b	61.46 ± 3.99 b
SA-Ricemate 300 ppm	12.50 ± 2.85 b	16.67 ± 1.41 b	17.71 ± 1.44 ab	58.63 ± 6.58 b	59.00 ± 5.86 b	67.24 ± 3.95 cb
Commercial elicitor	12.50 ± 2.14 b	17.50 ± 1.61 b	22.50 ± 1.12 b	57.13 ± 5.98 b	58.00 ± 3.60 b	58.38 ± 3.12 b
Copper hydroxide 77% WP	8.33 ± 1.04 a	10.42 ± 1.24 a	12.50 ± 1.20 a	72.42 ± 4.13 c	74.38 ± 2.88 c	76.88 ± 1.71 c
Control (water)	30.21 ± 4.10 c	40.63 ± 2.98 d	54.17 ± 3.75 d	0	0	0

^1^ Rice plants were treated by foliar sprays at 15, 30, and 45 DPS, with SA-Ricemate elicitor at different concentrations, commercial elicitor and copper hydroxide used as the positive control, and water used as the control. Rice leaves were inoculated with *Xoo* SUT1-121 strain at 50 DPS. ^2^ Disease severity was evaluated at 7, 14, 21 days post-inoculation. Each value represents a mean of five replicates. The mean in the column followed by the same letter (a, b, c, d) is a non-significant difference according to Duncan’s multiple range test at *p* = 0.05.

**Table 2:** Effectiveness of SA-Ricemate elicitor on the accumulation of endogenous SA in Rice leaf cv. KDML 105.

Treatment ^1^	Endogenous Salicylic Acid (µg g^−1^ of Fresh Weight) ^2^		Increase of SA Activity (%)
	Pre Inoculation	Post Inoculation 24 h	
SA-Ricemate 50 ppm	12.12 ± 0.17 a	17.60 ± 0.03 b	30.78 ± 1.87 b
SA-Ricemate 100 ppm	12.20 ± 0.18 a	18.46 ± 0.10 b	51.27 ± 3.10 b
SA-Ricemate 150 ppm	12.39 ± 0.14 a	18.34 ± 0.08 b	48.00 ± 4.42 b
SA-Ricemate 200 ppm	11.78 ± 0.26 a	18.14 ± 0.21 b	54.05 ± 6.24 b
SA-Ricemate 250 ppm	12.28 ± 0.22 a	18.28 ± 0.26 b	48.84 ± 4.42 b
SA-Ricemate 300 ppm	12.19 ± 0.21 a	18.46 ± 0.25 b	51.44 ± 4.64 b
Commercial elicitor	11.99 ± 0.38 a	16.61 ± 0.37 b	38.33 ± 4.17 b
Copper hydroxide 77% WP	11.69 ± 0.08 a	13.03 ± 0.32 a	5.19 ± 1.57 a
Control (water)	13.21 ± 0.10 a	13.89 ± 0.07 a	11.40 ± 2.57 a

^1^ Rice plants were treated by foliar sprays at 15, 30, and 45 DPS, with SA-Ricemate elicitor at different concentrations, commercial elicitor and copper hydroxide used as the positive control, and water used as untreated. Rice leaves were challenged with *Xoo* SUT1-121 strain at 50 DPS. ^2^ Endogenous salicylic acid was evaluated pre-inoculation and 24 h post-inoculation. Each value represents a mean of five replicates. The mean in the column followed by the same letter (a, b) is a non-significance difference according to Duncan’s multiple range test at *p* = 0.05.

## Data Availability

The data used to support the findings of this study are available from the corresponding author upon request.
